# Preparation of Chitosan from Brine Shrimp (*Artemia urmiana*) Cyst Shells and Effects of Different Chemical Processing Sequences on the Physicochemical and Functional Properties of the Product

**DOI:** 10.3390/molecules13061263

**Published:** 2008-06-06

**Authors:** Hossein Tajik, Mehran Moradi, Seyed Mehdi Razavi Rohani, Amir Mehdi Erfani, Farnood Shokouhi Sabet Jalali

**Affiliations:** 1 Department of Food Hygiene and Quality Control, Faculty of Veterinary Medicine, Urmia University, 1177, Urmia, Iran; 2 General Office of Veterinary, 45169-43374, Zanjan, Iran; 3 Department of Clinical Sciences, Faculty of Veterinary Medicine, Urmia University, 1177, Urmia, Iran.

**Keywords:** *Artemiaurmiana* cyst shells, chitosan, physicochemical characteristics, functional properties

## Abstract

Chitosan (CS) was prepared from *Artemia urmiana* cyst shells using the same chemical process as described for the other crustacean species, with minor adjustments in the treatment conditions. The influence of modifications of the CS production process on the physiochemical and functional properties of the CS obtained was examined. The study results indicate that *Artemia urmiana* cyst shells are a rich source of chitin as 29.3-34.5% of the shell’s dry weight consisted of this material. Compared to crab CS (selected as an example of CS from a different crustacean source) *Artemia* CS exhibited a medium molecular weight (4.5-5.7 ×10^5^ Da), lower degree of deacetylation (67-74%) and lower viscosity (29-91 centiposes). The physicochemical characteristics (e.g., ash, nitrogen and molecular weight) and functional properties (e.g., water binding capacity and antibacterial activity) of the prepared *Artemia* CSs were enhanced, compared to control and commercial samples, by varying the processing step sequence.

## Introduction

Chitosan (CS) and its derivatives are examples of value-added materials. They are produced from chitin, which is a natural carbohydrate polymer found in the skeleton of crustaceans, such as crab, shrimp and lobster, as well as in the exoskeleton of marine zooplankton spp., including coral and jellyfishes. Insects, such as butterflies and ladybugs, also have chitin in their wings and the cell walls of yeast, mushrooms and other fungi also contain this substance [1,2]. Industrial-scale CS production involves four steps: demineralization (DM), deproteinization (DP), decoloration (DC) and deacetylation (DA) [1,2]. Despite the widespread occurrence of chitin in nature, presently crab and shrimp shells remain the primary commercial sources. 

*Artemia* spp. (Crustacea, Anostraca), also known as brine shrimp, are typical inhabitants of extreme saline biotopes [3]. *Artemia* populations are found in about 500 natural salt lakes scattered throughout the tropical, subtropical and temperate climatic zones, along coastlines as well as inland [4]. In its natural environment at certain times of the year, *Artemia* produces cysts that float on the water surface. The cyst has shell and membranous coverings over the embryo which consists of three layers. The outer alveolar layer, a hard lipoproteinous layer, consists of lipoproteins impregnated with chitin and haematin, which serve as a protection layer for the embryo against mechanical disruption and UV radiation [4]. Urmia Lake is one of the biggest natural *Artemia* habitats in the world and it appears to be the only reservoir of the bisexual Old World *Artemia urmiana* [5]. The average number of cyst L^-1^ in Urmia Lake was 13 during 2003 and 11 during 2004 [5]. Almost all the *Artemia* cyst shells are currently discarded as a waste product after hatching and release of the free-swimming nauplii (first larval stage of *Artemia*). 

The objectives of the present study were to prepare chitosan from *Artemia* cyst shells and to evaluate the various changes caused by the sequential preparation processes (DP, DM, DC, and DA steps) used to prepare CS this source and to determine whether such modifications have any effect on yield, physicochemical (ash, moisture, nitrogen contents, molecular weight, viscosity, degree of deacetylation and color) and functional (water binding capacity, fat binding capacity and antibacterial activity) properties of the resulting CSs. Comparisons have also been made between CS obtained from the *Artemia* and commercial CS samples (Sigma Chemical Co., St Louis, MO, USA).

## Results and Discussion

**Figure 1:**
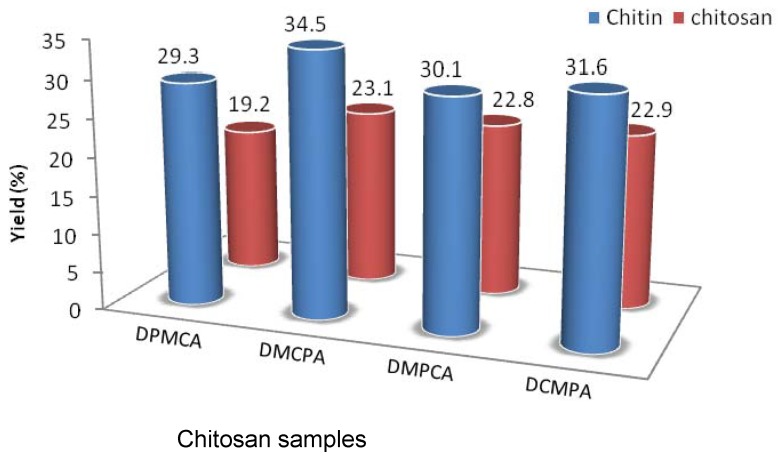
Chitin and chitosan production yield from *A. urmiana* cyst shells.

**Figure 2:**
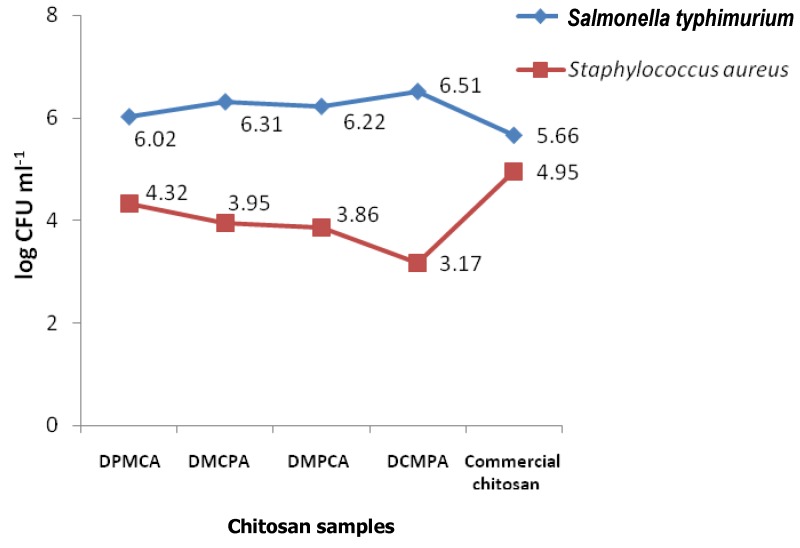
Antibacterial activity of four *Artemia* and commercial chitosan samples [0.1% (w/v)].

The present work represents the first attempt to investigate various physicochemical and functional properties of *Artemia urmiana* chitosan. The variation in physicochemical and functional properties of *Artemia* CS with changes in the four sequential processes of preparation was investigated. The results are shown in Figure 1 and Figure 2 and Table 1, Table 2 and Table 3.

### Effects of sequential process modifications on CS yield, moisture, ash and nitrogen contents

Figure 1 presents the percentage yields of chitin and CS from *Artemia* cysts obtained in this study. The different CS, labeled DPMCA, DMCPA, DMPCA and DCMPA, were prepared by changing the order of the four sequential preparation processes. For example, DPMCA denotes sequential steps of deproteinization + demineralization + decolorization + deacetylation. DPMCA represents the traditional processing method and was selected as the control sample. 

The yields depended on the CS extraction method, as DMCPA gave the highest chitin and CS yield (34.5 and 23.1%, respectively) and DPMCA gave the lowest (29.3 and 19.2%). However, the results indicated that when decoloration and demineralization steps were performed before demineralization and deproteinization, CS production was slightly increased. It has been repeatedly reported that the content of chitin in the shell waste of crustacean varies widely depending on the peeling conditions during processing, as well as the species [6,7,8]. Crustacean shells contain chitin amounts ranging from 13 to 42%, which in the case of crab (13 to 26%) is lower that in the case of shrimp (14 to 42%) and krill (34 to 49%) [8]. In comparison to commercial resources of chitin, *Artemia* *urmiana* had high levels of chitin contents, similar to krill.

The results of this work demonstrated that there was no significant difference in the % moisture (1.0-1.3%) between the four CSs prepared from *Artemia* (Table 1). The prepared CSs showed a relatively lower moisture content, compared to the commercial crab CS control sample. Since CS is a hygroscopic polymer [9], it is possible that the commercial samples were affected by moisture absorption during storage [10]. The moisture adsorption may be important by affecting water holding capacity of CSs, when it comes to its processing and applications [7].

Crustacean exoskeletons contain large amounts of calcium carbonate, depending on the source [6,7,11]. Some residual ash in CSs may affect final product qualities, including solubility and consequently contributing to lower viscosity [10]. As shown in Table 1, all CS samples had an excellent low ash content, ranging from 0.19 to 0.51%, indicating the effectiveness of the DM step in removing minerals. A high quality grade of CS should have less than 1% of ash content [12]. It has been reported Tolaimatea *et al.* [11], that lobster shell has a higher calcium content (30.54 %), whereas squid pens contain only 1.06 wt% of calcium. Our results (Table 1) show that the *Artemia* *urmiana* CSs contained a substantially lower amount of ash, which was consistent with the amount (4.05 wt%) reported previously [13]. Squid pens, similar to *Artemia* cysts, are very low in calcium content and it seems that a demineralization step is not necessarily required for these species [7]. The elimination of this step in the CS preparation should reduce the cost of processing and may also reduce the acid hydrolysis of the chitin that occurs during processing. The larger mineral content also causes an increase in the gas emissions. No and Meyers [12] have shown that the nitrogen content of CSs from various sources ranged from 7.06 to 7.97%. In this study, the nitrogen content of the CS products was in the 7.32-7.51% range. 

**Table 1:** % Ash, moisture and nitrogen of *A. urmiana* and commercial chitosan samples.

Chitosan samples ^a^	Ash %	Moisture%	N %
DPMCA	0.32± 0.02	1.2±0.19	7.51± 0.15
DMCPA	0.51±0.03	1.2±0.30	7.47± 0.07
DMPCA	0.25±0.01	1.3±0.29	7.48± 0.10
DCMPA	0.19±0.01	1.0±0.35	7.32± 0.90
Sigma^ b^	1.18±0.03	3.5±0.11	8.3± 0.90

Mean ± standard deviation of triplicate determinations, on a dry basis. ^a^ Abbreviations are as in Figure 1; ^b ^Commercial crab chitosan sample.

### Effects of sequential process modifications on CS Viscosity, MW and DD

The viscosity, degree of deacetylation (DD) and viscosity–average molecular weights resulting from the various CS preparation methods are shown in Table 2. Among the four samples and the commercial CS used in this study, DCMPA showed the highest viscosity (91cP) while the control sample (DPMCA) had the lowest viscosity, which suggests a decrease of MW. 

**Table 2:** MW, viscosity and DD of *A. urmiana* and commercial chitosan samples.

Chitosan samples ^a^	MW^ b^ ( X 10^5^)(Da)	Viscosity (cP)	DD^ c^ (%)
DPMCA	4.5±0.15	29± 0.25	70± 0.9
DMCPA	4.9±0.20	46±0.76	71± 1.3
DMPCA	4.6±0.09	35±0.50	67±1.2
DCMPA	5.7±0.14	91±0.11	74±1.2
Sigma ^d^	0.7±0.04	352± 0.15	79±0.7

Mean ± standard deviation of triplicate determinations, on a dry basis. ^a^ Abbreviations are as in Figure 1;^ b^ Molecular Weight; ^c^ Degree of Deacetylation; ^d^ Commercial crab chitosan sample.

This was in contrast to the findings of No and Meyers [12], who demonstrated that the viscosity of CSs varied considerably, from 60 to 5110 cP, depending on the species and preparation methods used. In our study, significant differences were found between the viscosity of DCMPA samples (91cP) and commercial CS (352cP), which was approximately sevenfold higher than average of *Artemia* CS. The viscosity obtained with the DCMPA method was 2-3-fold higher than with the DMCPA and DPMCA methods, respectively. Compared to other crustaceans [2], the viscosity of CS obtained from *Artemia* was lower. Lower viscosity of chitosan limits its applicability as a thickening and suspending agent for medical, cosmetic and food applications.

In the present study, MW ranged from 4.5- 5.7 ×10^5^ Da (Table 2). This range is considered suitable for several commercial applications [14]. Several factors during commercial production, including high temperature, concentration of alkali, reaction time, previous treatment of the chitin, particle size, chitin concentration, dissolved oxygen concentration and shear stress may influence the MW of CSs [15,16]. The molecular weights of CSs also affect their biological activities. For instance, CSs with molecular weights within the 5–20 kDa range, exhibited greater biological activities than total CS [17]. Similarly, significant correlations have been shown between molecular weight and viscosity (r = 0.903, P < 0.05) of six commercial CSs [14].

The DD is an important parameter affecting solubility, chemical reactivity, and biodegradability. Depending on the source and preparation procedure, DD may range from 30% to 95% [18]. This study (Table 2) revealed that, DD was ≥70% for all CSs except for DMPCA, which had a DD of 67%. The results from the present work showed that the *Artemia* cyst CS had a lower degree of acetylation than crustacean CSs [10]. A lower degree of acetylation reduces the amount of positively charged groups available for flocculating a negatively charged material e.g., bacteria [19].

A study by No *et al.* [14], reported a positive correlation between nitrogen and DD of six commercial CS samples and suggested that DD can be accurately estimated by measurement of nitrogen content. In the present work, DCMPA showed maximum DD (74%), compared with other samples, except for commercial CS. As shown in Table 2, reversing demineralization by decoloration or demineralization by deproteinization had no significant effects on DD percentage. Apart from the DMPCA method, degradation of chitin molecules during deacetylation decreased the molecular weights of both commercial and *Artemia* CSs samples and increased the DD levels (Table 2). As demonstrated by No *et al*. [20] elimination of the deproteinization step yields a CS with comparable nitrogen content and lower degree of DD, but higher molecular weight and viscosity than those of CSs prepared from DP for 5-30 min.

### Effects of sequential process modifications on CS WBC, FBC and Color

Water Binding Capacity (WBC), Fat Binding Capacity (FBC) and color of different samples of CS of *A. urmiana* and commercial CS are listed in Table 3. According to Rout [21] WBC for CS ranges between 581 to 1150% with an average of 702%. Those values were in agreement with results of this study, where WBC of the four CSs ranged from 654 to 721%, with an average of 609 %. A similar result has been reported by Cho *et al.* [22] but No *et al*. [14] reported lower results of 355 - 611%. 

The highest WBC was observed for DCMPA (721%), followed by DMPCA, DMCPA and DPMCA (701, 685 and 654%, respectively). As shown in Table 3, reversing the sequence of steps had marked effects on WBC. An increase in WBC was observed when demineralization was conducted prior to deproteinization followed by deacetylation, whilst this was not detected when deproteinization was performed prior to demineralization, followed by deacetylation. A similar result has been reported by Rout [21] in crawfish. He also reported that the process of decoloration causes a decrease in WBC of CS than those of unbleached crawfish CS. As illustrated in Table 3, there was no significant difference between the WBC of samples, except for commercial crab CS, which revealed a lower WBC (535%) than *Artemia* CS samples. No *et al.* [20] reported that the physicochemical characteristics of chitin and CS influence their functional properties, which differ with species and preparation procedures. Knorr [23] noticed that differences in WBC between chitinous polymers possibly were due to dissimilarities in crystallinity, differences in the amount of salt forming groups, and the residual protein content of the products.

The fat binding capacity of four *Artemia* and commercial CSs was measured using olive oil. As shown in Table 3, FBC of *Artemia* CS ranged from 420 to 481%. No significant differences in FBC were observed among the four CSs tested. The range of FBC found in our study (314 - 535%) was slightly similar to that reported by Cho *et al.* [22] and slightly higher than that (217 - 403%) explained in [14]. Several studies reported a correlation between physicochemical and functional properties of CS. Cho *et al*. [22] found that both WBC and FBC had a significant positive correlated with ash (r = 0.81, 0.80), and negatively correlated with bulk density (r = -0.98, -0.95). FBC showed a correlation with molecular weight (r = 0.802, P = 0.055) and with viscosity (r = 0.834, P < 0.05) [14] However, in another study, ash showed no correlation with either WBC (r = -0.239) or FBC (r = -0.100) [12].

Chitosan isolated from most sources is perfectly white and soft [21], unlike that isolated from *Artemia* cysts, which varied from little brownish to light yellow (Table 3.). Either an incomplete decoloration step or the presence of considerable quantities of astaxanthin in *Artemia* cysts, as compared with other crustacean’s exoskeletons, are suggested as the reasons for this. According to Abdou *et al*. [6] and No *et al*. [20] some sources like squid pen contain small amounts of carotenoids. 

**Table 3:** WBC, FBC and color of *A. urmiana* and commercial chitosan samples.

Chitosan samples^ a^	WBC^ b^ (%)	FBC^ c^ (%)	Color
DPMCA	654±11.52	420.3±12.32	little brownish
DMCPA	685±15.01	454.9±5.89	light yellow
DMPCA	701±7.6	481.2±21.68	little brownish
DCMPA	721±9.3	450.3±19.71	little brownish
Sigma^ d^	535±15.1	471.5±9.20	white

Mean ± standard deviation of triplicate determinations, on a dry basis. ^a^ Abbreviations are as n Figure 1; ^b^ Water Binding Capacity; ^c ^Fat Binding Capacity; ^d^ Commercial crab chitosan sample.

### Effects of sequential process modifications on CS antibacterial activity

Antimicrobial activities of CS against various pathogens and food-spoilage microorganisms were investigated for the purpose of their application in food processing and preservation [25,26,27]. As illustrated in Figure 2, 0.1% DCMPA, with average MW of 5.7 ×10^5^ D, had a stronger antibacterial effect on *Staphylococcus aureus* than DPMCA, DMCPA, DMPCA and commercial CSs, respectively. It reduced the initial number (8.12 log CFU mL^-1^) of *S. aureus* up to 3.17 log CFU mL^-1^. Moreover, addition of CS (0.1%) reduced the number of *Salmonella typhimurium* from 8.52 to 6.02 log CFU mL^‑1^. According to Yang *et al*. [28] cells of *S. aureus* were found most susceptible to CS and CS derivatives in the late-exponential phase, followed by late stationary phase and mid-exponential phase.

Many authors [29,30,31,32,33,34] have suggested that CSs inhibit the growth of most bacteria, although their inhibitory effects differ with molecular weight and the particular bacterial sp. CS generally showed stronger bactericidal effects for gram-positive bacteria than gram-negative bacteria [20,34,35]. Their result supports the findings of this study, suggesting that different CSs at 0.1% concentration had less antibacterial activity against gram negative than gram-positive bacteria. However, for CS with different MW, the antimicrobial effect on gram-positive bacteria was strengthened as the MW increased. In contrast, in gram negative bacteria the antibacterial activity was enhanced as the MW decreased [36]. The poor inhibitory effect of CS on *S.* typhimurium can possibly be attributed to the relatively small number of charged amino groups in the molecules [37]. However, according to Zheng and Zhu [36] increasing the concentration of CS led to an increased antimicrobial effect. When the concentration reached 1.0%, the inhibition rate reached 100% for both *E. coli* and *S. aureus*. 

## Experimental

### Preparation of Artemia cyst shells

Brine shrimp (*Artemia urmiana*) cysts were obtained fresh from Sehrdaroo Co. Ltd, Urmia, Iran. Cysts were cleaned from debris, sand and salt crystals and consequently hatched according to the standardized conditions described by Sorgeloos *et al*. [4]. Following the hatching process, the cyst shells were collected from the top of the hatching containers and processed as follows: (i) density separation in brine, (ii) washing several times in fresh water, (iii) density separation in fresh water, (iv) dried at 60°C overnight in a forced air oven (v) ground to a powder (500 g) with a cutting mill and (vi) storage at –5 ± 2°C for as long as needed.

### Preparation of chitosan

Chitin extraction from *Artemia* cyst shells was carried out as described previously for other crustacean shells by an alkali-acid treatment with minor modifications of the treatment conditions [10]. Four *Artemia* CSs labeled DPMCA, DMCPA, DMPCA and DCMPA were prepared by changing of the order of the four sequential preparation processes. For example, DPMCA denotes sequential steps of deproteinization + demineralization + decolorization + deacetylation. DPMCA was taken as the traditional processing method (control sample).

Depending upon the production order, samples (referred to as cyst shells, demineralized or decolorized samples) were deproteinized by treating with 1.2 N sodium hydroxide for 2.5 hr at 70-75 °C (10 mL g^-1^ of samples), demineralized at room temperature with 0.7 N hydrochloric acid (10 mL g^-1^ of samples) for 15 min and decolorized with acetone for 10 min and dried for 2 hr under hood, followed by bleaching with 0.32 % (v/v) solution of sodium hypochloride (containing 5.25% available chlorine) for 15 min at ambient temperature (15 mL g^-1^ of samples). After each step, the solid was filtered off, washed with distilled water to neutral pH. Chitin deacetylation was carried out at 15 psi/121 °C using 50% sodium hydroxide solution (13 mL g^-1^ of chitin) for 15 min. After this step, samples were filtered off, washed with distilled water to neutral pH and dried in an oven at 60 °C for 8 h. Commercial crab CS of Sigma was used as a control to compare with the *Artemia* CSs produced in this study.

### Determination of physicochemical and functional properties: yield, moisture, ash and nitrogen content

CS yield was determined by comparing weight measurements of the raw material and of the CS obtained after treatment. Moisture content of the samples was determined according to the standard method [38] with minor modification. Moisture of samples was determined by drying the samples at 60°C for 24 h or until the weights were constant. It was then calculated by percentage of weight loss comparing to the initial weight of the samples. Ash and nitrogen contents of CSs were measured according to a previously described procedure [38].

### Molecular Weight (MW), Viscosity and Degree of Deacetylation (DD)

For the determination of molecular weight (MW), CSs were dissolved in a solvent system, constituted of acetic acid (0.1 M), sodium chloride (0.2 M) and water. An Automated Solution Viscometer (Relative Viscometer Model Y501, Viscotek Corp., Houston, TX, U.S.A.) was used to measure the intrinsic viscosity [η]. The MW of CSs was calculated using classical Mark–Houwink relationship: [η] = KM^α^, where [η], K and α are intrinsic viscosity; 3.1×10^-3^ and 1.01, respectively [13]. The viscosities of the prepared CS samples determined with a Brookfield viscometer (Brookfield Engineering Laboratories). CS solution was prepared using 1% acetic acid at a 1% concentration on a dry basis. Measurement was made using a No. 5 spindle at 50 rpm on solutions at 25^o^C with values reported in centipoises (cPs) units. However, the DD percent was determined by a titration method in which CS was dissolved in 0.1% acetic acid to form a 0.01% solution. This was followed by titration with 0.0025 N potassium polyvinyl sulfate with 1% toluidine blue (TBO) as an indicator. The acetyl content of CS was measured from the amount of titrant used [39].

### Color, Water Binding Capacity (WBC) and Fat Binding Capacity (FBC)

Color evaluation of samples was conducted visually using a four member panel. WBC and FBC of chitosans were measured using the method of No *et al*. [14]. Briefly, the procedure was carried out by weighing a centrifuge tube containing 0.5 g sample, adding 10 mL of water or olive oil, and mixing on a vortex mixer for 1 min to disperse the sample. The contents were left at ambient temperature for 30 min with shaking for 5s every 10 min and centrifuged at 3200 rpm for 25 min. The supernatant was decanted and the tube was weighed again. WBC and FBC were calculated using following formula:

WBC (%) = [water bound (g)/sample weight (g)] × 100; FBC (%) = [fat bound (g)/sample weight (g)] × 100.

### Evaluation of antibacterial activity: preparation of organisms

Lyophilized cultures of *S. aureus* RTCC 1885 and *S.* *typhimurium* 138, phage type 2 (confirmed by the Pasteur Institute, Paris, France) was obtained from the Department of Food Hygiene and Quality Control, Faculty of Veterinary Medicine, University of Tehran, Tehran, Iran. Bacterial suspensions were prepared in Brain Heart Infusion (BHI) Broth and were cultured at 37 ±1°C for 24 h. Then, a second subculture was prepared and incubated for 18 h at 37 ±1°C. Bacterial suspensions adjusted to 10^8^ CFU mL^-1^ final cell concentrations according to spectrophotometer measurements at 600 nm. 

### Antibacterial screening

Four CS solutions as well as Sigma crab CS solution were prepared in 1% (v/v) acetic acid at a concentration of 1% (w/v). Each solution was dispersed in BHI broth to give a final CS concentration of 0.1% (w/w). Subsequently, each bacterium (25 μL) was inoculated into BHI broth (5 mL) containing the CS solution. Following bacterial inoculation, tubes incubated at 37 °C for 24 h. A 0.1 mL dilution of this broth was spread plated onto BHI agar and incubated at 37°C for 24 h for later colony counting.

### Statistical analysis

All experiments, except for yield and antibacterial screening, were carried out in triplicate and results were expressed as mean ± S.D. Analysis of variance was performed using SPSS statistical package program (SPSS 13.0 for Windows, SPSS Inc., Chicago, IL).
